# Is neural hyperpolarization by cathodal stimulation always detrimental at the behavioral level?

**DOI:** 10.3389/fnbeh.2014.00226

**Published:** 2014-06-27

**Authors:** Cornelia Pirulli, Anna Fertonani, Carlo Miniussi

**Affiliations:** ^1^Cognitive Neuroscience Section, IRCCS Istituto Centro San Giovanni di Dio FatebenefratelliBrescia, Italy; ^2^Neuroscience Section, Department of Clinical and Experimental Sciences, University of BresciaBrescia, Italy

**Keywords:** transcranial direct current stimulation, perceptual learning, metaplasticity, facilitation, cathodal tDCS, homeostasis, NIBS, neural noise

## Abstract

Cathodal transcranial direct current stimulation (c-tDCS) is usually considered an inhibitory stimulation. From a physiological perspective, c-tDCS induces hyperpolarization at the neural level. However, from a behavioral perspective, c-tDCS application does not always result in performance deterioration. In this work, we investigated the role of several important stimulation parameters (i.e., timing, presence of pauses, duration, and intensity) in shaping the behavioral effects of c-tDCS over the primary visual cortex. In Experiment 1, we applied c-tDCS at two different times (before or during an orientation discrimination task). We also studied the effects of pauses during the stimulation. In Experiments 2 and 3, we compared different durations (9 vs. 22 min) and intensities (0.75 vs. 1.5 mA) of stimulation. c-tDCS applied before task execution induced an improvement of performance, highlighting the importance of the activation state of the cortex. However, this result depended on the duration and intensity of stimulation. We suggest that the application of c-tDCS induces depression of cortical activity over a specific stimulated area; but to keep reactivity within given limits, the brain react in order to restore the equilibrium and this might result in increased sensitivity in visual performance. This is a further example of how the nervous system dynamically maintains a condition that permits adequate performance in different environments.

## Introduction

Transcranial direct current stimulation (tDCS) is a technique that allows the modulation of cortical excitability. A direct current of low-level intensity (~2 mA) is applied to electrodes that sit on the subject’s scalp. This current passes through the scalp and crosses the extra cortical layers to reach the cortex, modulating the membrane polarity of neurons within a region of underlying neural tissue. The low strength of the current is not able to induce depolarization at a threshold level of “inactive” neurons (i.e., inducing an action potential). However, if there is ongoing activity (i.e., background activity determined by state or task-induced activity), the change in membrane potential induced by tDCS can promote more effective “excitation” or “inhibition” in a polarity-specific manner (Creutzfeldt et al., 1962; Bindman et al., 1964; Stagg and Nitsche, 2011). These tDCS-induced changes in the neuronal threshold during stimulation result from changes in membrane permeability, including depolarization of the soma by anodal stimulation (a-tDCS) and hyperpolarization by cathodal stimulation (c-tDCS) (Liebetanz et al., 2002; Nitsche et al., 2003b,2004a,b). Polarization effects outlast the tDCS period (Nitsche and Paulus, 2000, 2001), and these after-effects are due to changes in receptor activity at the synaptic level, in addition to membrane polarity shifts (Nitsche et al., 2003a, 2004a,b).

From a behavioral standpoint, strong *a priori* assumptions are often made in neuromodulation studies using tDCS where physiological effects are directly mapped on to behavioral effects. The application of a-tDCS during a task is thought to induce facilitation, while c-tDCS is assumed to induce inhibition of performance. While the concept of a-tDCS facilitating and c-tDCS worsening performance seems well established, these effects are mainly valid for tDCS in the motor system (Nitsche et al., 2008, but see Wiethoff et al., 2014). Behavioral effects of tDCS have been identified in several functional areas, but the relationship between facilitation and inhibition is often quite complex (Jacobson et al., 2012; Miniussi et al., 2013). tDCS is thought to prime the behavioral system by increasing/decreasing cortical excitability and producing behavioral effects in the cognitive system. Nevertheless, the final effect depends on the stimulated area and on its involvement in a given task (i.e., the type of process and the final goal of the task) (Dockery et al., 2009; Nitsche and Paulus, 2011; Vallar and Bolognini, 2011; Berryhill and Jones, 2012; Jacobson et al., 2012; Tseng et al., 2012; Weiss and Lavidor, 2012; Filmer et al., 2013; Hsu et al., 2014; Nozari et al., 2014).

Several examples of contradictory final behavioral outcomes for c-tDCS can be found at different processing levels (Jacobson et al., 2012). Monti et al. (2008) found facilitation in a naming task using c-tDCS over Broca’s area. Antal et al. (2004b) found that c-tDCS applied to the visual middle temporal area improved performance in a visuomotor coordination task. In a different study from the same group (Antal et al., 2001) using a Gabor patch stimulus, only c-tDCS showed a significant change in static and dynamic contrast sensitivities. Vicario et al. (2013) showed that c-tDCS applied to the posterior parietal cortex enhanced temporal accuracy in a time reproduction task compared to sham stimulation (see also Dockery et al., 2009; Moos et al., 2012; Weiss and Lavidor, 2012; Filmer et al., 2013). Moreover, several studies reported no effect of c-tDCS on task performance (e.g., Kincses et al., 2004; Iyer et al., 2005; Sparing et al., 2008; Cerruti and Schlaug, 2009; Fertonani et al., 2010; Kraft et al., 2010). Contrasting effects of c-DCS have also been found at the neurophysiological level (see Matsunaga, 2004; Pellicciari et al., 2013). A magnetoencephalography study has reported that the two tDCS polarities induce the same cortical EEG power density (Venkatakrishnan et al., 2011). Antal et al. (2004a) showed that c-tDCS over the primary visual cortex (V1) decreased the amplitude of an early visual evoked potential (N70), whereas Accornero et al. (2007) reported the opposite result with an increased amplitude of the early P100 potential.

This complex pattern of results may be explained by the fact that the ability or the efficacy of tDCS to induce modifications of membrane polarity—and consequently behavioral performance—depends on several methodological and technical parameters: current density, duration, timing of application, and pauses between stimulations (for a review, see Brunoni et al., 2012). Clearly, all these methodological aspects will determine the “reaction of the brain” to the DC stimulation in relation to the subject’s state and task/protocol. As an example, the importance of duration and of the presence of pauses during the period of stimulation has been recently demonstrated by Fricke et al. (2011). These authors measured the motor evoked potential (MEP) amplitude after repeated tDCS. The authors found that 5 or 10 min of c-tDCS decreased excitability and suppressed MEP amplitude for 5 and 30 min, respectively, indicating the importance of tDCS duration. However, if a pause of 3 min was inserted between two stimulations of 5 min, there was an inversion of the effect, resulting in MEP amplitude enhancement (see also Monte-Silva et al., 2010). The stimulation intensity is also an important factor. Batsikadze et al. (2013) showed that 1 mA c-tDCS decreased MEP amplitude, but the application of 2 mA resulted in increased cortical excitability (see also Teo et al., 2011; Moos et al., 2012; Hoy et al., 2013). In addition, behavioral performance induced by tDCS depends on the timing of application in relation to task execution (Stagg et al., 2011; Pirulli et al., 2013; Fertonani et al., 2014). Several studies have shown that the same type of stimulation may have different behavioral effects (facilitation vs. inhibition vs. null-effect) depending on whether it is applied before or during the task execution.

Given the heterogeneity of the effects induced by c-tDCS, the aim of this work was to explore the outcome of applying c-tDCS on V1. We chose an orientation discrimination task (ODT) because it is a well established task to study visual perceptual learning (Vogels and Orban, 1985; Shiu and Pashler, 1992), and it has been showed to involves primarily V1 (Schoups et al., 2001; Li et al., 2004). Nevertheless it should be considered that we cannot be totally sure that stimulation delivered by tDCS is focalized only under the stimulation electrode (Miranda et al., 2006; Wagner et al., 2007). We applied c-tDCS before or during the execution of ODT, with or without pauses during stimulation, and at different intensities and durations. Based on the previous reports, our expectation was that the final outcome of the tDCS in terms of response facilitation or inhibition would depend on the interaction between the brain state and when and how c-tDCS was applied. Therefore, c-tDCS would not necessarily induce a univocal behavioral inhibition. Given that the brain is constructed to keep certain important parameters within given limits, the brain would react proportionally when it is shifted from these limits in order to restore the equilibrium, as suggested by the concept of homeostasis (see Bernard, 1878 and Cannon, 1929 in Cooper, 2008). Therefore, an initial down-regulation induced by an inhibitory stimulation, given for a longer time, at higher intensity can be reverted, rendering the involved neurons more easily responsive. Nevertheless to explain the effects of tDCS during a behavioral task it has been proposed that a neural noise framework should be consider (see non-linear systems and stochastic resonance; Miniussi et al., 2013), suggesting that the outcome of applying tDCS depends on the noise present in the system and the level of tDCS and task -induced activity, rather than solely on the stimulation polarity.

## Materials and methods

### Orientation discrimination task

**Figure 1:**
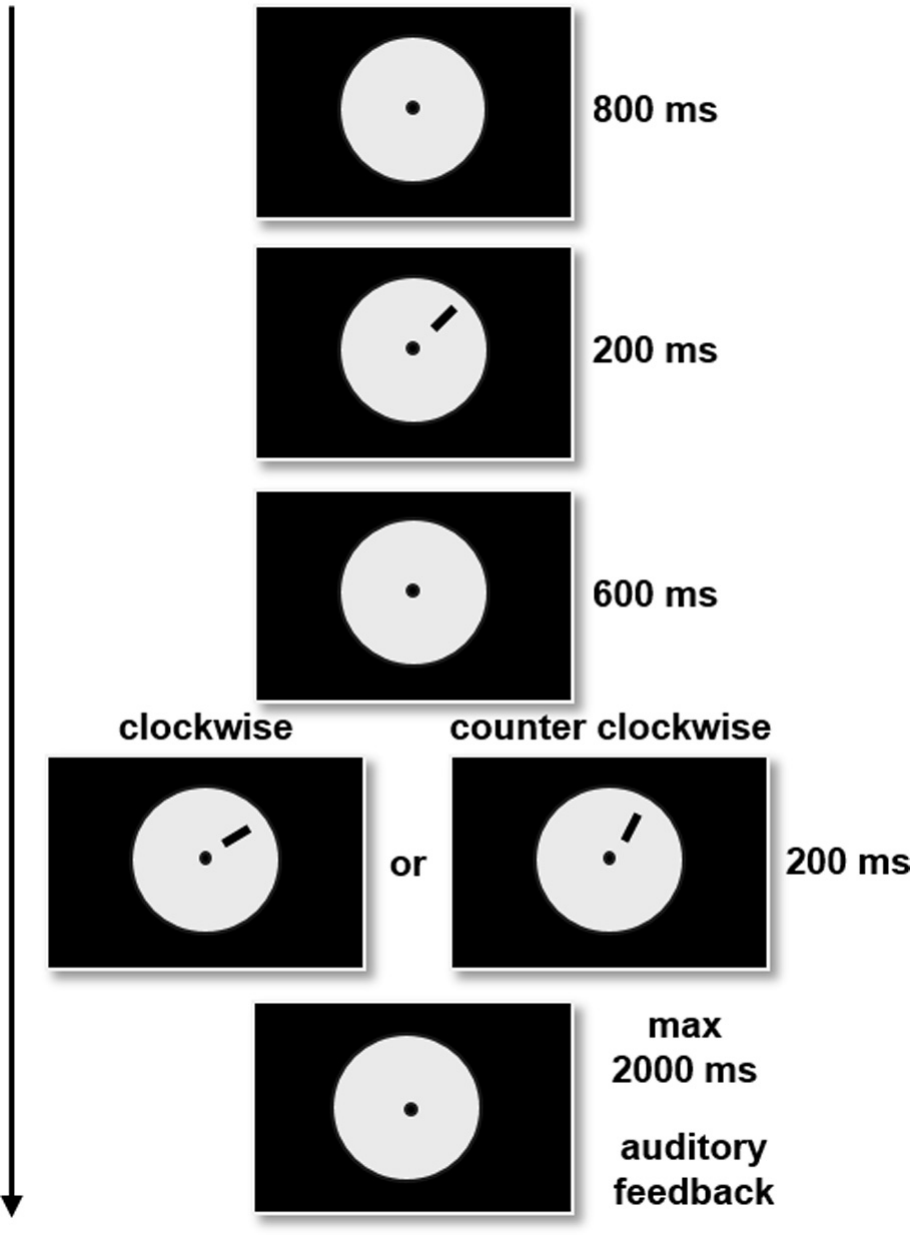
**Example of an ODT trial with the reference and target stimuli presented in the upper right hemifield**. The line can turn clockwise or counter clockwise. The subjects were asked to decide whether the presented stimulus was tilted clockwise or counter clockwise relative to the previously presented stimulus.

We chose an ODT that is a widely studied VPL task and involves V1 neurons (Vogels and Orban, 1985; Shiu and Pashler, 1992). This task has been previously described in detail by our group (Fertonani et al., 2011; see Pirulli et al., 2013). Given the ODT characteristics, is likely that a local (i.e., V1) circuit of neuronal populations is dedicated to execute the task, nevertheless we cannot exclude that tDCS effects are due to a more complex neuronal network, involving others parietal areas. Briefly, throughout the experiment, participants were comfortably seated in an armchair in a quiet, dimly illuminated room. The subjects had to decide as quickly and accurately as possible whether the presented stimulus (a target line) was tilted clockwise or counter clockwise relative to the previously presented stimulus (reference line) (see trial structure in Figure 1). After each response an auditory feedback informed the subjects about the correctness of their responses (an high tone indicated the right response while a low tone the wrong response).

Each block of the ODT consisted of 64 trials and lasted approximately 4 min. The ODT consisted of 5 experimental blocks plus a training block. The training block contained 8 trials and an increased rotation angle between the two stimuli (10° clockwise or counter clockwise). In Experiments 1 and 2, the angular difference between the reference and the target was ±1.10, 1.21, 1.33, and 1.46°. All of the experimental parameters were balanced and randomized between blocks. In Experiment 3 (control experiment), the task was made easier by replacing the smallest degree of rotation (1.10°) with 1.60° (1.21, 1.33, 1.46, and 1.60°). All of the other task characteristics were unchanged, except for the presence of a baseline block before the stimulation.

### Transcranial direct current stimulation

tDCS was delivered by a battery-driven current stimulator (Eldith-Plus, NeuroConn GmbH, Ilmenau, Germany) through a pair of saline-soaked surface sponge electrodes. The “active” electrode (16 cm^2^) was placed over the occipital cortex in the area corresponding to V1, which was defined as 10% of the nasion-inion distance above the inion (mean position = 3.5 ± 0.2 cm above the inion). The reference electrode (60 cm^2^) was fixed extra-cephalically on the right arm. The electrodes were kept in place with elastic bands, and an electro-conductive gel was applied under the electrodes to help reduce impedance to the electrical current. When tDCS was applied, the polarity of the active electrode over V1 was always cathodal. For active tDCS, the current was ramped up over 8 s (fade-in phase), held constant for the experimental time, and then ramped down over 8 s (fade-out phase). In the sham c-tDCS, the current was ramped up (8 s) and down (8 s) and stayed at level for 15 s.

#### Experiment 1—“timing and pauses”

**Figure 2:**
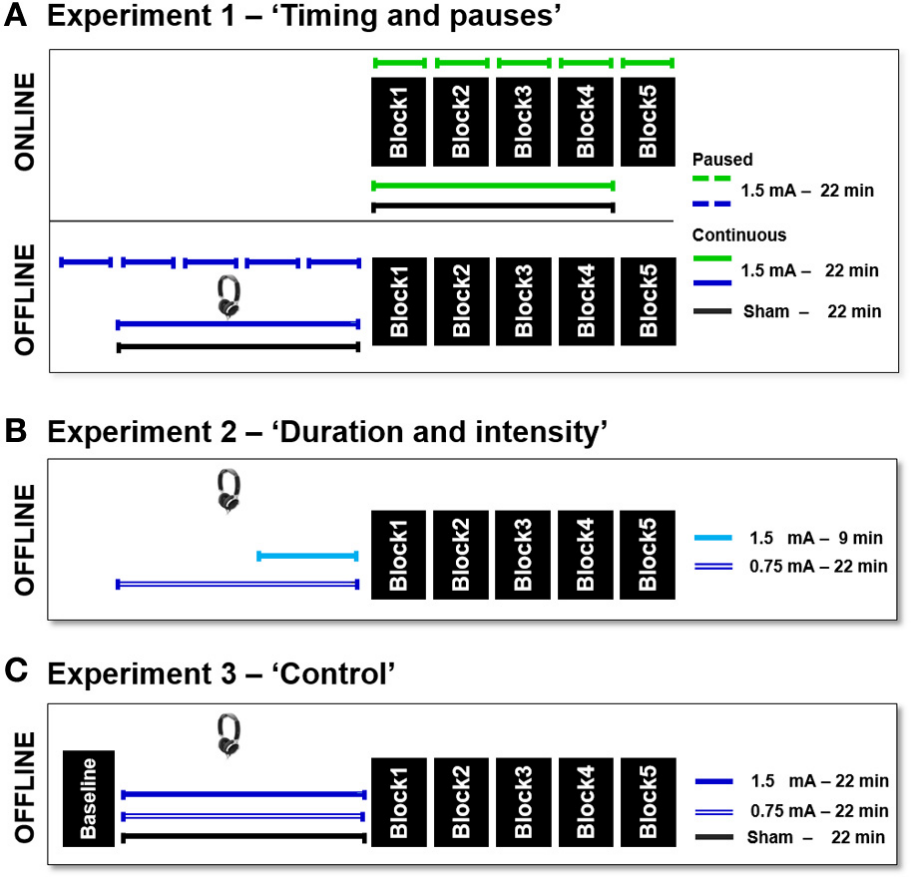
**Experimental procedure. (A)** Experiment 1: in the top panel, the green lines represent the online conditions. In the bottom panel, the blue lines represent the offline conditions. The dashed lines represent the paused stimulations, whereas the continuous lines the continuous stimulations. The sham condition is shown in black lines. **(B)** Experiment 2 (offline conditions): the short turquoise line represents 1.5 mA for 9 min, and the double blue line represents 0.75 mA for 22 min. **(C)** Experiment 3 (offline conditions): the blue line represents 1.5 mA for 22 min, the double blue line represents 0.75 mA for 22 min, and the black line represents sham for 22 min.

In this experiment, we investigated the effect of the timing of stimulation during task execution, either online or offline (before task execution), and the presence of pauses during the stimulation (intervals of 2 min between blocks). In all conditions, we applied c-tDCS for 22 min at 1.5 mA (current density of the active electrode 0.094 mA/cm^2^ of the reference 0.025 mA/cm^2^) as shown in Figure 2A. In the continuous stimulation, current was administered without pauses, while in the paused stimulation condition, the stimulation was turned on at the beginning of each experimental block and maintained until the end of the block. c-tDCS was applied for approximately 4 min during each of the 5 experimental blocks, with 2 min of pauses between blocks (i.e., 4 min of stimulation—2 min of pause—4 min of stimulation, and so on). In the online condition, the stimulation was applied during the task execution. In the offline condition, stimulation was applied before task execution while the subjects were listening to an audio book played on an audio device, maintaining the same time intervals used in the online condition. The duration of the entire experimental session was approximately 30 min for the online conditions and approximately 60 min for the offline conditions. The procedure is described in Figure 2A.

#### Experiment 2—“duration and intensity”

In this experiment, we investigated the effect of the duration and intensity of stimulation for the offline condition, as shown in Figure 2B. Subjects were stimulated for 9 min at an intensity of 1.5 mA or for 22 min at an intensity of 0.75 mA (current density of the active electrode 0.047 mA/cm^2^ of the reference 0.013 mA/cm^2^). Data were compared with those for 22 min of stimulation at an intensity of 1.5 mA in the offline condition collected in Experiment 1 (see Figure 2B).

#### Experiment 3—control experiment

In Experiment 3, we corroborated the effects of the stimulation intensity for the offline protocol with a modified experimental design (see the description of the ODT task, above). We facilitated the ODT and added a baseline block before the beginning of the offline stimulation. The stimulation was applied continuously for 22 min with an intensity of 1.5 or 0.75 mA (see Figure 2C).

#### Sensation questionnaire

In all of the experiments, at the end of the experimental session, we asked all subjects to complete a questionnaire (Fertonani et al., 2010) about the tDCS-induced sensations that they experienced during the different conditions so that we could evaluate if different stimulation protocols (e.g., active vs. sham) induced different sensations.

### Subjects

A total of 139 healthy subjects participated in the three experiments. All of the participants were right-handed except for 6 subjects tested in Experiment 3, who were equally distributed in the experimental groups. All participants had normal or corrected-to-normal vision. Subjects with a history of seizures, implanted metal objects, heart problems or any neurological disease were not recruited. Moreover, subjects who had a task performance below chance (no learning) were excluded from the study. Based on these criteria, 17 participants were excluded. The remaining 122 subjects (61 males, mean age ± standard deviation 22.0 ± 2.9 years; range 19–33 years) participated in the experiments.

#### Experiment 1—“timing and pauses”

Seventy-two subjects were assigned to one of the five groups stimulated for 22 min at 1.5 mA: online paused (14 subjects, 7 males; 21.7 ± 2.6 years), offline paused (14 subjects, 7 males; 21.6 ± 2.6 years), online continuous (10 subjects, 5 males; 21.7 ± 0.8 years), offline continuous (10 subjects, 5 males; 23.0 ± 3.6 years) and placebo stimulation (sham, 24 subjects, 12 males; 21.7 ± 3.6 years). In the sham group, 14 subjects were stimulated online and 10 subjects were stimulated offline. The data for the online conditions were collected in a previous experiment (Fertonani et al., 2011; for details see Pirulli et al., 2013).

#### Experiment 2—“duration and intensity”

We tested two additional groups (20 Subjects): 10 subjects (5 males; 22.1 ± 2.0 years) stimulated for 9 min at 1.5 mA (offline-9 min) and 10 subjects (5 males; 21.4 ± 2.0 years) stimulated for 22 min at 0.75 mA (offline-0.75 mA). We compared these two groups with the offline and sham groups of Experiment 1.

#### Experiment 3—control experiment

We tested three new groups (30 Subjects) stimulated for 22 min at 0.75 mA (10 subjects, 5 males, 23.0 ± 4.4 years), 1.5 mA (10 subjects, 5 males, mean age 20.8 ± 1.8 years) and sham (10 subjects, 5 males; 23.6 ± 4.1 years).

The study was approved by the Ethics Committee of the IRCCS Centro San Giovanni di Dio Fatebenefratelli, Brescia, Italy. Safety procedures were used in accordance with non-invasive brain stimulation indications (Iyer et al., 2005; Poreisz et al., 2007; Rossi et al., 2009), and written informed consent was obtained from all participants prior to the beginning of the experiments.

### Data analysis

The average orientation sensitivity was calculated as a d prime value (d′) from measurements of the hit rate and false-alarm rate, for each subject and each block separately for each stimulation condition. We have chosen the d′ as a measure of accuracy because it is roughly invariant when response bias is manipulate, whereas simple indexes such as proportion correct don’t have this property. As a first index of learning rate, we analyzed the relationship between d′ values and block numbers using linear regression analysis. This analysis allowed us to associate a slope value with each subject. A second index called the “learning index” was calculated, for Experiment 3, by subtracting the mean baseline dx02032; value from the mean dx02032; value of block 5 for each subject.

The Kolmogorov-Smirnov test confirmed the normality of the distribution of all data (dx02032; values, slope, learning index), and subsequently data were analyzed using a repeated-measures analysis of variance (ANOVA). The data sphericity was tested using the Mauchly test, where appropriate. When the test results were statistically significant, the data were corrected using the Huynh-Feldt correction. The effect size is reported using the partial Eta squared value. A *p*-value < 0.05 was considered significant for all statistical analyses. For multiple comparisons, we used Fisher’s Least Significant Difference (LSD) method to test our specific “a priori” hypotheses (i.e., to compare different timings of application, intensities and durations). For all other comparisons, the *p*-values were corrected using a Tukey correction.

Data from the sensations induced by c-tDCS were analyzed using the Kruskal–Wallis one-way analysis of variance and, subsequently, with multiple comparisons.

## Results

### Orientation sensitivity—d′

#### Experiment 1—“timing and pauses”

We performed a repeated-measure ANOVA with *block* (from 1 to 5) as a within-subjects factor and *stimulation* (online paused, online continuous, offline paused, offline continuous, and sham) as a between-subjects factor. We observed a significant main effect for *block* [*F*_(4, 268)_ = 24.105; *p* < 0.001; η ^2^_P_ = 0.265] and *stimulation* [*F*_(4, 67)_ = 3.272; *p* = 0.016; η ^2^_P_ = 0.163]. The interaction between *block* and *stimulation* was not statistically significant [*F*_(16, 268)_ = 1.005; *p* = 0.452].

For *block*, multiple *post-hoc* comparisons revealed a statistically significant difference between block 1 and blocks 2, 3, 4, and 5 (all *p* < 0.01), between block 2 and blocks 4, and 5 (all *p* < 0.001), and between block 3 and block 5 (*p* = 0.043).

**Figure 3:**
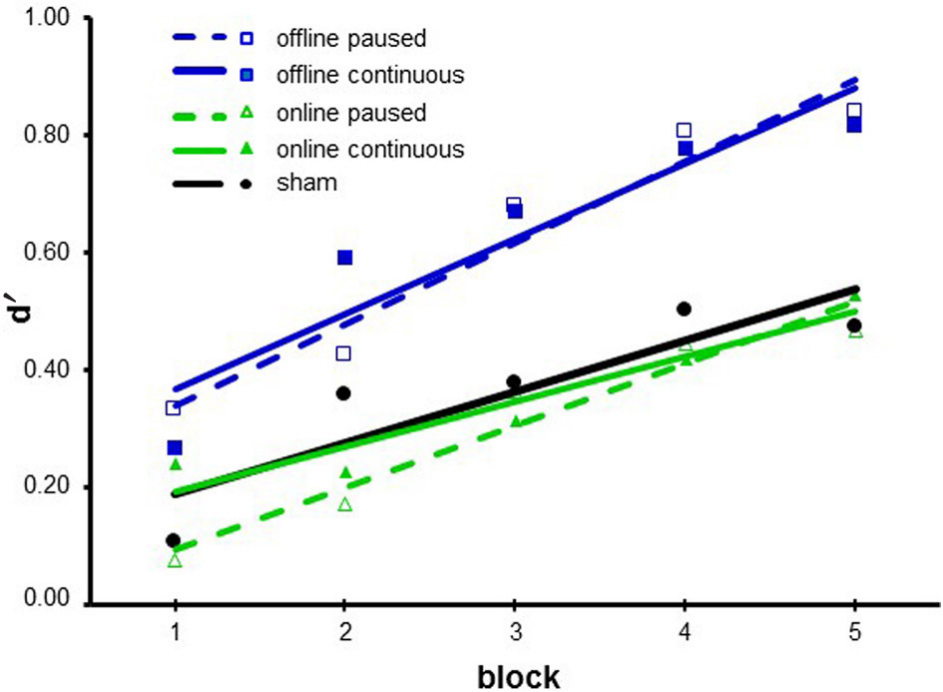
**Results of Experiment 1**. Data are presented as the mean d′ values. The lines represent the fit of each condition. The blue lines represent the offline conditions, and the green lines represent online conditions. The dashed lines represent the paused stimulations, whereas the continuous lines represent the continuous stimulations. The sham condition is shown in black.

For *stimulation*, multiple *post-hoc* comparisons revealed that the offline paused (mean d′ ± standard error of the mean—s.e.m. = 0.615 ± 0.122) and offline continuous (0.623 ± 0.114) conditions were significantly different from the online paused (0.304 ± 0.121), online continuous (0.346 ± 0.118), and sham (0.368 ± 0.083) (all *p* < 0.05) conditions (see Figure 3).

These results support the initial hypothesis that c-tDCS, applied before the ODT, modulates behavior, while c-tDCS applied during the task does not modify the final outcome. We found an improvement in the subject’s accuracy when c-tDCS was applied offline.

These data highlight the absence of a difference between the conditions with or without pauses. Confirmation was obtained with an ANOVA with *block* (from 1 to 5) as a within-subjects factor and *timing* (online vs. offline) and presence of *pauses* (paused vs. continuous) as between-subjects factors. We observed a significant main effect of *block* [*F*_(4, 176)_ = 18.438; *p* < 0.001; η ^2^_P_ = 0.295] and *timing* [*F*_(1, 44)_ = 11.026; *p* = 0.002; η ^2^_P_ = 0.200]. The factor *pauses* was not statistically significant [*F*_(1, 44)_ = 0.077; *p* = 0.782]. No interaction was statistically significant. For *block*, multiple *post-hoc* comparisons revealed a statistically significant difference between block 1 and blocks 3, 4, and 5 (all *p* < 0.001) and between block 2 and block 3 (*p* = 0.042), 4 (*p* < 0.001), 5 (*p* < 0.001) (see Figure 3).

Having verified that the presence of pauses during stimulation does not influence the effect of stimulation, we collapsed the two online conditions (continuous and paused) and the two offline conditions (continuous and paused) into one online and one offline condition (hereafter, all conditions with the initial parameters, i.e., 22 min duration and 1.5 mA intensity, will be called “online” and “offline”).

#### Experiment 2—“duration and intensity”

We performed a repeated-measure ANOVA with *block* (from 1 to 5) as a within-subjects factor and *stimulation* (online, offline, offline-9 min, offline-0.75 mA, and sham) as a between-subjects factor. We observed a significant main effect for *block* [*F*_(4, 348)_ = 20.286; *p* < 0.001; η ^2^_P_ = 0.189] and *stimulation* [*F*_(4, 87)_ = 4.727; *p* = 0.002; η ^2^_P_ = 0.163]. The interaction between *block* and *stimulation* was not statistically significant [*F*_(16, 348)_ = 1.512; *p* = 0.092].

For *block*, multiple *post-hoc* comparisons revealed a statistically significant difference between block 1 and blocks 2, 3, 4, and 5 (all *p* < 0.001), between block 2 and blocks 4, and 5 (all *p* < 0.001), and between block 3 and block 5 (*p* = 0.003).

**Figure 4:**
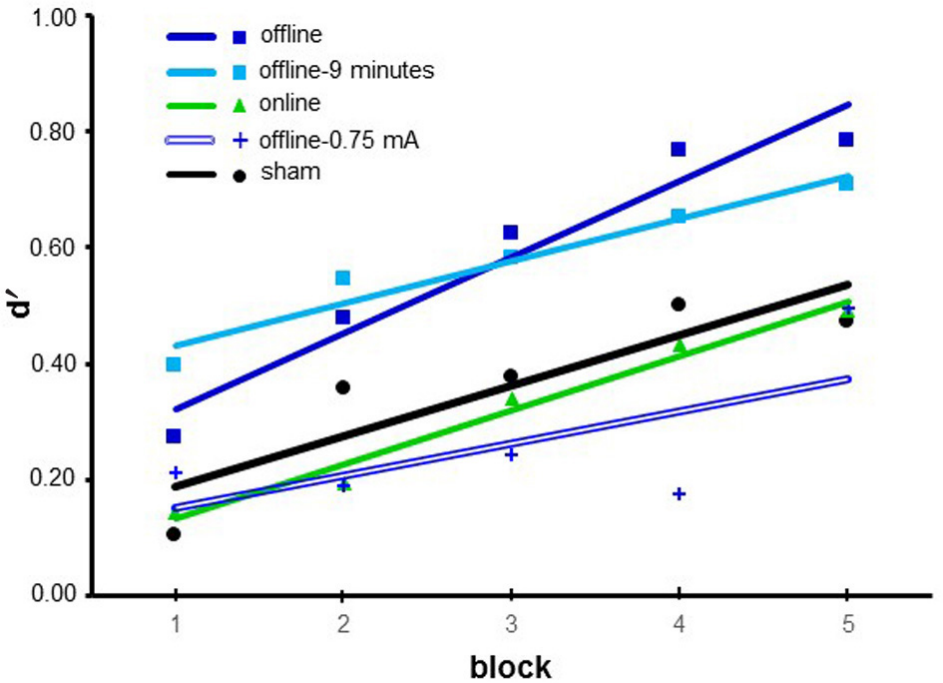
**Results of Experiment 2**. Data are presented as the mean d′ values. The lines represent the fit of each condition. The blue line represents the offline 1.5 mA for the 22 min condition, the green line represents the online 1.5 mA for the 22 min condition, the turquoise line represents offline 1.5 mA for the 9 min condition, and the double blue line represents the offline-0.75 mA for the 22 min condition. The sham condition is shown in black.

For *stimulation*, multiple *post-hoc* comparisons revealed that offline (0.618 ± 0.085) was different from sham (0.368 ± 0.083), online (0.321 ± 0.086) and offline-0.75 mA (0.264 ± 0.109). Moreover, offline-9 min (0.577 ± 0.123) was different from online and offline-0.75 mA and showed a marginally significant difference from sham (*p* = 0.069, all other *p* < 0.05) (see Figure 4).

#### Experiment 3—control experiment

We performed a repeated-measure ANOVA with *block* (from baseline to 5) as a within-subjects factor and *stimulation* (offline, offline-0.75 mA, and sham) as a between-subjects factor. We observed a significant main effect for *block* [*F*_(5, 135)_ = 7.843; *p* < 0.001; η ^2^_P_ = 0.225] and an interaction between *block* and *stimulation* [*F*_(10, 135)_ = 1.980; *p* = 0.128]. The factor *stimulation* was not statistically significant [*F*_(2, 27)_ = 1.434; *p* = 0.256].

For *block*, multiple *post-hoc* comparisons revealed a statistically significant difference between block baseline and blocks 1, 2, 3, 4, and 5 (all *p* < 0.01). The interaction between *block* and *stimulation* revealed that stimulation influences the block trend. In the offline condition, block baseline was different from blocks 1, 2, 3, 4, and 5; block 1 was different from blocks 4 and 5; and block 2 was different from block 5. In the offline-0.75 mA condition, block baseline was different from block 4. In the sham condition, block baseline was different from blocks 1 and 5 and block 1 was different from block 2.

A One-Way ANOVA on block baseline showed that in this block, d′ was not different between the stimulation conditions [*F*_(2, 27)_ = 0.064; *p* = 0.938].

**Figure 5:**
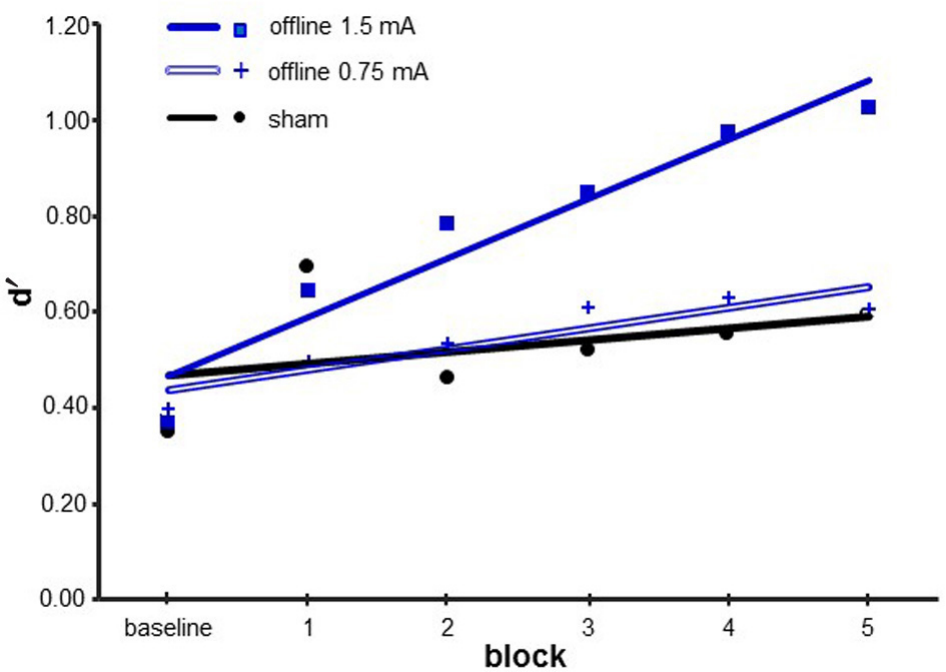
**Results of Experiment 3**. Data are represented as the mean d′ values. The lines represent the fit of each condition. The blue line represents the offline 1.5 mA for the 22 min condition. The double blue line represents the offline 0.75 mA for the 22 min condition. The sham condition is shown in black.

In Experiment 3, the presence of a block of baseline allowed us to show a different rate of learning between the stimulation conditions. For this purpose, we executed two different analyses. A One-Way ANOVA on slope [*F*_(2, 27)_ = 4.630; *p* = 0.019; η ^2^_P_ = 0.255] demonstrated that offline was different from sham (*p* = 0.008) and 0.75 mA (*p* = 0.027). A One-Way ANOVA on the learning index [*F*_(2, 27)_ = 3.658; *p* = 0.039; η ^2^_P_ = 0.213] showed that offline was different from sham (*p* = 0.034) and 0.75 mA (*p* = 0.022) (see Figure 5).

#### Sensations induced by different conditions

In the tDCS sensation questionnaire (Fertonani et al., 2010), each participant reported having tolerated the stimulation without discomfort. The results of the questionnaire are reported in Table 1 (Experiments 1 and 2) and Table 2 (Experiment 3). In Experiments 1 and 2, the analysis did not reveal any differences between the stimulations for pain, burning, heat, iron taste, and fatigue sensations. The analysis demonstrated a statistically significant difference between stimulations with respect to itchiness [*H*_(6, *N* = 92)_ = 17.382, *p* = 0.008] and pinching [*H*_(6, *N* = 92)_ = 18.974, *p* = 0.004]. Subsequently, multiple comparisons were performed for these two sensations. For itchiness, offline continuous was significantly different (*p* = 0.03) from sham, whereas for pinching, online paused and offline paused were significantly different from sham (respectively *p* = 0.03 and *p* = 0.01). The subjective influence on performance was equal for all the stimulations. In Experiment 3, the analysis did not reveal any difference between the stimulation conditions in the perceived sensations.

**Table 1:** **Transcranial direct current stimulation (tDCS)-induced sensations—Experiments 1 and 2: mean intensity of the sensations reported by subjects after tDCS and the percentage of subjects who reported each sensation**.

**Stimulation condition**		**Itchiness**	**Pain**	**Burning**	**Heat**	**Pinching**	**Iron taste**	**Fatigue**	**Effect on performance**
Sham	Intensity	0.3	0.0	0.2	0.2	0.5	0.0	0.3	–
	Subjects (%)	25	0	17	17	38	4	25	–
Online paused	Intensity	0.6	0.1	0.3	0.1	1.4	0.1	0.2	0.1
	Subjects (%)	50	7	21	7	93	7	14	14
Online continuous	Intensity	0.9	0.2	0.3	0.3	1.0	0.0	0.2	0.2
	Subjects (%)	50	20	30	30	70	0	20	20
Offline paused	Intensity	0.6	0.3	0.6	0.1	1.5	0.1	0.4	–
	Subjects (%)	50	14	43	7	93	7	21	–
Offline continuous	Intensity	1.1	0.2	0.7	0.2	1.2	0.0	0.1	–
	Subjects (%)	90	20	60	20	80	0	10	–
Offline 9 min	Intensity	1.2	0.1	0.2	0.1	1.1	0.0	0.1	–
	Subjects (%)	80	10	20	10	80	0	10	–
Offline 0.75 mA	Intensity	0.7	0.1	0.3	0.3	1.0	0.0	0.0	–
	Subjects (%)	70	10	30	30	100	0	0	–

*Sensation intensity was evaluated on a 5-point scale: 0 none, 1 mild, 2 moderate, 3 considerable, and 4 strong. The column “Effect on performance” indicates the subjective feelings of the participants relative to the effect of the tDCS-induced sensation on performance (valid only for the online condition)*.

**Table 2:** **Transcranial direct current stimulation (tDCS)-induced sensations—Experiment 3 (See Table 1 description)**.

**Stimulation condition**		**Itchiness**	**Pain**	**Burning**	**Heat**	**Pinching**	**Iron taste**	**Fatigue**
Sham	Intensity	0.4	0.0	0.3	0.1	0.6	0.0	0.0
	Subjects (%)	40	0	30	10	60	0	0
1.5 mA	Intensity	0.6	0.0	0.4	0.3	1.1	0.1	0.0
	Subjects (%)	50	0	30	30	90	10	0
0.75 mA	Intensity	1.3	0.1	0.1	0.2	0.9	0.0	0.3
	Subjects (%)	70	10	10	10	70	0	30

## Discussion

In this work, we demonstrated that c-tDCS, which is often considered to be inhibitory at the behavioral level, can actually induce facilitatory effects, enhancing subjects’ performance. In the Experiment 1, we showed that the effects of c-tDCS are dependent on the timing of stimulation. Only c-tDCS before the task induced an improvement of performance. Moreover, in this experiment, we applied 22 min of stimulation interspersed by pauses of 2 min and compared this protocol to a continuous protocol. We found that short pauses do not play any role in shaping the final outcome. In Experiment 2, we applied offline stimulation and found that 9 min of c-tDCS facilitated the performance only slightly less. Instead, current density induced a facilitatory effect in our protocol. Finally, Experiment 3 confirmed the importance of current density and showed that the facilitatory effect is not due to skill differences between subjects (i.e., differences in accuracy) because all the groups had the same performance at baseline.

The presence of short pauses (2 min) during c-tDCS, both in the online and in the offline conditions, did not influence the results. In previous works, it has been shown that inter-stimulation intervals determine the effects of c-tDCS on the motor cortex (Monte-Silva et al., 2010; Fricke et al., 2011). These previous studies found that varying the length of the pause between two stimulations modified the effects on cortex excitability. Fricke et al. (2011) demonstrated that short inter-stimulation intervals (3 min) induced an inversion of the inhibitory effect of c-tDCS, resulting in MEP amplitude enhancement. Monte-Silva et al. (2010) showed that the inhibitory effects of c-tDCS were more efficacious if a second period of stimulation was applied during the after-effects of the first stimulation with an interval of 3 or 20 min. However, with long inter-stimulation intervals, the c-tDCS-induced inhibitory after-effects were diminished. In Experiment 2 of our study, we confirmed that c-tDCS applied before the task induces a significant improvement in performance, regardless of the presence of pauses during stimulation. A possible explanation for these partially contrasting data could be the differences in stimulation parameters (i.e., the intensity and number of pauses\blocks of stimulation). A higher intensity of stimulation (1.5 mA) may induce a stable excitability shifts in relation to the execution of the task, and therefore, the additional presence of pauses might not further affect the cortical state (see Batsikadze et al., 2013). Similarly the presence of successive multiple pauses\blocks of stimulation might decrease further changes of the cortical state through adaptation.

During the online application of c-tDCS, while the subjects were performing the ODT, we expected a worsening of performance. However, it was difficult to observe a decline in subject accuracy in our learning task because subjects could not have a performance level lower than chance (“floor effect”). This result suggests that the behavioral level of task performance at baseline is a key factor in determining a null effect of c-tDCS. Additionally, compensatory networks can be activated during stimulation (Sack et al., 2005), and therefore, a functional compensation might intervene, maintaining behavior after neuronal challenge (O’Shea et al., 2007). Nevertheless, the absence of inhibition by online c-tDCS is in line with previous data (Jacobson et al., 2012).

Our most important result involves the fact that c-tDCS applied before the task at 1.5 mA induced a facilitatory effect on subjects’ accuracy during the ODT (see also Dockery et al., 2009; Moos et al., 2012; Weiss and Lavidor, 2012; Filmer et al., 2013). Most studies that have applied 1 mA c-tDCS in the motor system found decreased cortical excitability; this has also been shown with a decrease in MEP amplitude (see Nitsche and Paulus, 2011; Medeiros et al., 2012). However, these physiological effects are not linear, but they seem to depend on several parameters. In a recently published work, Batsikadze et al. (2013) showed that 20 min of c-tDCS at 2 mA applied to the motor cortex significantly increased MEP amplitudes, while 1 mA of the same stimulation decreased cortico-spinal excitability. This result highlights that an increase in intensity (2 mA) and duration (20 min) of stimulation induces an opposite outcome compared to standard parameters. Batsikadze et al. (2013) suggested that this result might be due to the direction of plasticity from the amount of neuronal calcium influx caused by the stimulation protocol: whereas low postsynaptic calcium enhancement induced by low-intensity c-tDCS causes long-term depression, higher intensity c-tDCS induces a large calcium increase, resulting in long-term potentiation (LTP) (Cho et al., 2001; Lisman, 2001; Batsikadze et al., 2013). Our data are in line with this result; nevertheless, at a systems level, different intensities of stimulation might induce different adaptive responses by the brain, as discussed below.

Importantly, our stimulation was applied before the task execution. The time at which the stimulation is applied has been investigated at the behavioral level in the motor (Stagg et al., 2011) and visual domains (Pirulli et al., 2013). Stagg et al. (2011) showed that a-tDCS on the motor cortex has opposite effects if applied during or before a sequence—a motor learning task. However, the effects of c-tDCS do not seem to be timing dependent. c-tDCS application during or before an explicit motor learning task induces a slowing in reaction time (Stagg et al., 2011). Nevertheless, in a previous work (Pirulli et al., 2013), we demonstrated that the same type of current can induce different effects on visual performance depending on the state of activation of the cortex.

Here, we demonstrated that the application of c-tDCS before the VPL induces an improvement in performance that is not present if c-tDCS is applied during the execution of the task. Therefore, c-tDCS causes different effects depending on the state level of the neurons at the moment of stimulation (Dockery et al., 2009; Tseng et al., 2012; Weiss and Lavidor, 2012; Filmer et al., 2013; Hsu et al., 2014; Nozari et al., 2014). While an online effect would rely mainly on the depolarization or hyperpolarization induced by tDCS interacting with task execution (see Miniussi et al., 2013), an offline protocol would rely more on a change in the state of the stimulated area induced by the stimulation i.e., a shift in the input strength needed for the final response (see Figure 5 in Miniussi et al., 2013). Thus, all these aspects, which can be defined as “relatively simple” technical parameters, influence brain activity in response to exogenous stimulation.

Artificially altering neuronal function can trigger homeostatic changes at the synaptic level (Turrigiano and Nelson, 2004). For example, if a synapse is constantly over-inhibited, there can be a compensatory increase in receptor activity at the postsynaptic membrane, termed up-regulation. Homeostatic plasticity is a fundamental physiological mechanism that maintains neural functions within predefined optimal ranges (Bienenstock et al., 1982; Turrigiano and Nelson, 2004; Abraham, 2008). The basis of homeostatic plasticity is that the threshold for LTP induction is not stable but varies depending on previous neuronal activity induced by non-invasive stimulation (Ziemann and Siebner, 2008; Siebner, 2010). Therefore, the application of tDCS before and during the task of interest may result in different functional states. The application of c-tDCS before the execution of the task would lower the level of postsynaptic neural activity, causing a decrease in the threshold for the induction of successive facilitatory mechanisms. The induced neural modification could then facilitate LTP-like mechanisms and consequently induce an improvement in behavioral performance (Bienenstock et al., 1982; Abraham, 2008). Homeostatic mechanisms can stabilize cortical excitability within a range (Siebner et al., 2004). Thus, an initial down-regulation induced by c-tDCS can be reverted. Applying this theory to our data, pre-conditioning the V1 cortex with 22 min of inhibitory stimulation could render neurons that are involved in task execution more easily excitable. In classical studies, a release phenomenon (rebound) has been found at the end of the cathodal DC (Creutzfeldt et al., 1962). However, the timing of this inversion might depend, at least in part, on stimulation parameters like intensity and duration. Indeed, stimulating offline with a different duration or intensity changes the behavioral effects. Our data highlighted the fact that the final behavioral response, obtained by applying tDCS, depends on the history of the stimulated area (i.e., metaplasticity). This has been previously demonstrated in animal studies in which the direction and magnitude of synaptic plasticity depends on the previous history of postsynaptic activity (Huang et al., 1992; Wang and Wagner, 1999).

From a physiological perspective, c-tDCS over the scalp can induce a hyperpolarization at the soma of perpendicularly oriented neurons (Jefferys, 1981; Bikson et al., 2004). However, we are measuring behavior and therefore should consider a network level of reasoning rather than just a cellular one and present data testify the complexity of a neural network response. Indeed, changes in hyperpolarization alter the sensitivity of the entire system and therefore its response threshold, but these changes are ultimately expressed on subject performance. The final response to the task depends on the strength of the signal and on the signal-to-noise ratio, where the signal is the neural activity operational to the task and the noise is random neural activity. Clearly, in this case, the signal-to-noise ratio relies on a system that has changed its state after c-tDCS, a system that has adapted to exogenous stimulation. Adaptation occurs when receptors\neurons change their sensitivity to a stimulus. In the visual system, adaptation to reduced light intensity increases the visual system’s ability to detect a stimulus. In the same way, the reduced cortical activity induced by c-tDCS causes an improved threshold for orientation sensitivity (d′) by reducing background noise. Therefore, an inhibitory stimulation may increase the signal-to-noise ratio in the system and facilitate perceptual learning (Miniussi et al., 2013).

In conclusion, the present data show that an inhibitory stimulation does not always induce a deterioration in performance. The effects of c-tDCS should be considered in relation to the timing and the application parameters that will alter the state of the cortical network carrying out a task. We suggest that when applying c-tDCS before a task, it is necessary to consider the involvement of cognitive and non-cognitive adaptation mechanisms. The application of a tDCS protocol that induces a depression in cortical activity over a specific stimulated area might result in increased sensitivity in visual performance. This is a further example of how the nervous system maintains a dynamic state to maintain performance in different environments.

### Conflict of interest statement

The authors declare that the research was conducted in the absence of any commercial or financial relationships that could be construed as a potential conflict of interest.
